# Whole-course application of dexmedetomidine as an adjuvant to spinal-epidural anesthesia for cesarean section: A randomized, controlled trial

**DOI:** 10.1016/j.heliyon.2023.e23534

**Published:** 2023-12-10

**Authors:** Yang-yang Wu, Zheng Fang, Kun-shan Liu, Meng-di Li, Xin-qi Cheng

**Affiliations:** a Department of Anesthesiology, First Affiliated Hospital of Anhui Medical University, 218 Jixi Road, Hefei, Anhui, China; b Department of Anesthesiology, People's Hospital of Linquan, 109 Tongyang Road, Linquan, Anhui, China

**Keywords:** Dexmedetomidine, Spinal anesthesia, Ropivacaine, Patient-controlled analgesia, Visual analog scale

## Abstract

**Background:**

Dexmedetomidine is known to prolong the analgesic duration of spinal anesthesia, but it can be challenging to achieve further extension without opioids. Therefore, this study aimed to investigate a novel analgesic strategy using dexmedetomidine as an adjuvant to spinal-epidural anesthesia for elective cesarean surgery.

**Methods:**

The study was a randomized, double-blind, controlled trial conducted at a single center. Sixty parturients who underwent elective cesarean were randomly assigned to either group C or group D. Group D received an intrathecal injection of 12.5 mg ropivacaine and 5 μg dexmedetomidine followed by continuous epidural patient-controlled analgesia (PCA) infusion with a total volume of 100 ml, containing 0.2 % ropivacaine and 0.5 μg/kg dexmedetomidine. Group C received an intrathecal injection of 12.5 mg ropivacaine with an equivalent saline placebo followed by a similar PCA infusion, containing 0.2 % ropivacaine and an equivalent saline placebo.

**Results:**

The primary outcome was visual analog scale score on movement at 24 h after surgery. The results showed that the rest and motion pain scores in group D were significantly lower than those in group C at 6 h, 12 h, and 24 h after surgery (*P* < 0.05), with the differences at 24 h were 5.0 (5.0, 5.0)in group D versus 5.0 (5.0, 6.0) in group C (*P* = 0.04). Additionally, the time to the first PCA in group D was significantly longer than that in group C (*P* < 0.05), as well as the time of sensory and motor recovery.

**Conclusions:**

Whole-course application of dexmedetomidine as an adjuvant to spinal-epidural anesthesia could effectively extend the analgesic duration of ropivacaine to 24 h following elective cesarean surgery.

## Highlights

• Dexmedetomidine is known to prolong the analgesic duration of spinal anesthesia.
• Adding dexmedetomidine to spinal-epidural anesthesia could extend analgesic duration to 24 h following cesarean surgery.
• This novel analgesic strategy was no increasing in the risk of adverse effects.

## Abbreviations

ASA: American Society of Anesthesiologists
VSA: visual analog scale
PCA: patient-controlled analgesia
CSEA: combined spinal-epidural anesthesia
BMI: body mass index
NIBP: non-invasive blood pressure
SpO_2_: percutaneous arterial oxygen saturation
HR: heart rate
RR: respiratory rate

## 1Introduction

Combined spinal-epidural anesthesia (CSEA) is commonly used for elective cesarean section due to its minimal effect on the fetus and good safety and controllability. To enhance the quality of anesthesia, reduce the dose of intrathecal local anesthetics, and minimize side effects [[1], [2], [3]], a combination of intrathecal local anesthetics and adjuvants is often administered. Adjuvants may include opioids and other drugs such as dexmedetomidine, clonidine, and magnesium sulfate. However, it's important to note that opioids acting on the spinal cord can sometimes lead to adverse reactions, such as respiratory depression, nausea, and vomiting [4,5].

Ropivacaine is a new and long-lasting local anesthetic that can effectively alleviate pain during labor and postoperative periods. It has the advantage of lower toxicity to the cardiovascular and central nervous systems, shorter duration of motor blockade, and faster recovery of motor function [6,7]. Dexmedetomidine, a highly selective α_2_ receptor agonist, provides multiple pharmacological effects including sedation, analgesia, and anti-sympathetic effects. Studies have shown that using dexmedetomidine as a local anesthetic adjuvant provides better anesthesia effects than intrathecal local anesthetics alone in obstetric surgeries [[8], [9], [10], [11]]. When ropivacaine is used with dexmedetomidine during a cesarean section, its pain relief only lasts 6 h post-surgery [12]. This makes it challenging to extend the pain relief. To address this issue, we conducted a prospective, randomized, double-blind, controlled study to evaluate a novel analgesic strategy that utilizes dexmedetomidine as an adjuvant after spinal epidural anesthesia in patients undergoing elective cesarean surgery.

## 2Methods

### 2.1Study design

After approval by the Medical Ethics Committee of People's Hospital of Lin-quan, Anhui Province (No. SL-YX2021-02) and registration with the Chinese Clinical Trial Registry (ChiCTR) (www.chictr.org, No. ChiCTR 2100050473). The study included 80 women undergoing elective cesarean section surgery between October 2021 and January 2022. Participants met specific criteria and signed an informed consent form.

To be eligible for this study, participants had to meet the following criteria: (1) aged between 18 and 40 years old; (2) have an American Society of Anesthesiologists (ASA) physical status classification of I or II; (3) have a singleton pregnancy. Those who met any of the following criteria were excluded from the study: (1) having pregnancy-induced hypertension requiring treatment; (2) having a history or current presence of cardiac, respiratory, hepatic, and/or renal failures; (3) being in active labor, having a twin pregnancy, placenta previa, or abruptio placentae; (4) having a preoperative heart rate (HR) less than 50 beats/min with cardiac conduction or rhythm abnormalities; (5) having an allergy to α2 adrenergic agonists or local anesthetics; (6) having contraindications to neuraxial block; (7) body mass index (BMI) > 38 kg/m^2^; (8) having extreme heights of less than 140 cm or greater than 180 cm.

### 2.2Randomization and blinding

Sixty parturients were randomly divided into Group D (12.5 mg ropivacaine + 5 μg dexmedetomidine) and Group C (12.5 mg ropivacaine + equivalent saline placebo) in a 1:1 ratio. Those in Group D received continuous epidural patient-controlled analgesia (PCA) with a total volume of 100 ml, consisting of 0.2 % ropivacaine and 0.5 μg/kg dexmedetomidine. Meanwhile, those in Group C received a similar PCA infusion with 0.2 % ropivacaine and an equivalent saline placebo. All PCA was set up with a continuous infusion rate of 2 ml/h, a single bolus dose of 2 ml, and a lockout time of 15 min.

The computer system generated a random number table, which was used for randomization. The solution for spinal-epidural anesthesia was mixed by an anesthesiologist who was not involved in any further anesthesia work or evaluation. One anesthesiologist who was unaware of the grouping performed all anesthesia procedures for the parturients and did not participate in postoperative evaluations. All postoperative data were assessed and documented by a third anesthesiologist who was also unaware of the group allocation.

### 2.3Anesthetic procedure

Upon entering the operating room, the patient's vital signs including non-invasive blood pressure (NIBP), percutaneous arterial oxygen saturation (SpO_2_), heart rate (HR), and respiratory rate (RR) were monitored. Lactated Ringer's solution was infused through a vein in the forearm at a rate of 8–10 mL/kg. The patient was positioned on their left side and a lumbar puncture was performed at the L2-L3 intervertebral space. An epidural injection of 12 mg of ropivacaine (Aspen Pharmacare Australia Pty Ltd, H20140763) with 4 μg of dexmedetomidine (Yangtze River Pharmaceutical Co. Ltd, H20183219) or a placebo was given after the cerebrospinal fluid was obtained. The success of the injection was determined using the loss of resistance technique. A catheter was inserted into the epidural space and oxygen was administered through a mask. If the patient's blood pressure or heart rate showed significant changes, ephedrine or atropine was administered accordingly. The anesthesiologist was not aware of the solution injected in this trial. was performed.

### 2.4Outcomes

The primary outcome measure was the visual analog scale (VAS) scores for motion 24 h after surgery.

The secondary outcomes included: (1) resting and motion VAS scores within 72 h postoperatively; (2) usage of the PCA, including the first time to PCA, The PCA frequency within 24 and 48 h postoperatively; (3) block duration, including the time to onset of sensory block (defined as the time from injection of the drug to the time the sensory level reached T10), time to maximal sensory level (defined as the time from injection of the drug to the time the sensory level reached T6 or above), time to sensory recovery (defined as the time from the end of surgery to the time of recovery of pain and touch sensation in the lower extremities), and time to motor recovery (defined as the time when the patient was able to achieve a modified Bromage scale of 0); (4) recovery time, including time to gastrointestinal function recovery, time to first ambulation, and length of hospital stay; and (5) incidence of adverse events, including postoperative nausea and vomiting, dizziness, constipation, abdominal distension, shivering, and urinary retention.

### 2.5Statistical analysis

We used G*Power (Version 3.1.9.4) from Dusseldorf, Germany to determine the sample size needed for our study. In our preliminary observation, we studied ten patients (five in each group) and found that the mean VAS on motion at 24 h postoperatively was 3.1 with a standard deviation of 1.0 in group D, while it was 4.0 with a standard deviation of 1.3 in group C. We aimed for a two-sided α-level of 0.05 and a statistical power of 80 %. We also allowed for a 5 % loss to follow-up. Based on these factors, we calculated that we would need 30 patients in each group (a total of 60 patients) to detect a difference of means of 50 %. We also expected an SD within groups of 30 %.

The statistical analysis was performed using SPSS 24.0 software. Normally distributed measurement data are expressed as mean ± standard deviation and *t-*test is used for between-group comparison; Non-normally distributed measurement data are expressed ass median (p25, p75), using two independent samples of Mann-Whitney U-tests. The generalized estimating equation method was used to compare the trend variances between groups. Count data were expressed as the number (rate), and the comparison between groups was performed using the chi-square test, with a significance level of α = 0.05.

## 3Results

**Fig. 1:**
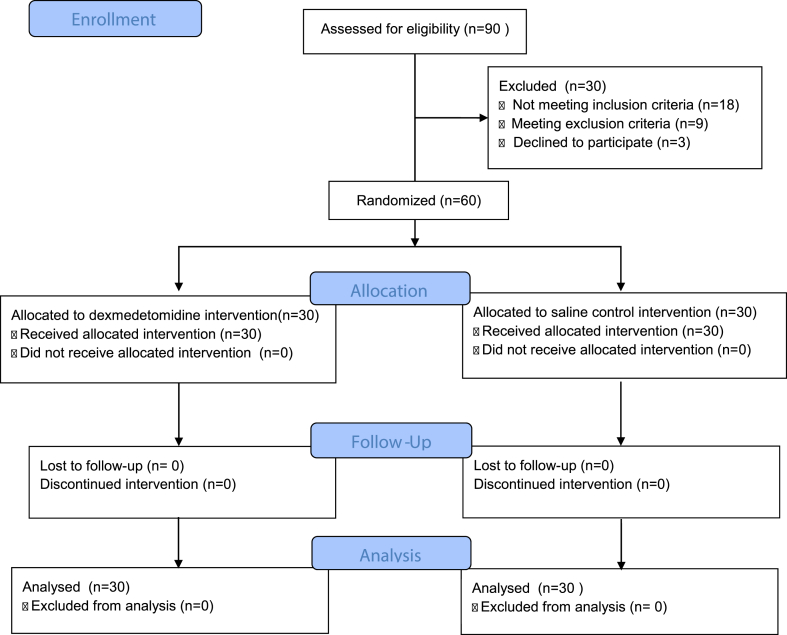
Flow diagram.

We enrolled 90 pregnant women, but 18 of them didn't meet the criteria for the study, 9 were excluded, and 3 declined to participate. In the end, we randomly assigned 30 women to Group C and 30 to Group D (as shown in Fig. 1). Population demographics are shown in Table 1. There were no significant differences between the two groups in terms of demographic and obstetric data (*P* > 0.05).

**Table 1:** Demographic characteristics.

	Group D (n = 30)	Group C (n = 30)	P value
Age (years)	30.0 ± 5.4	29.0 ± 4.6	0.177
Weight (kg)	73.8 ± 10.1	76.0 ± 11.4	0.457
Height (cm)	160.8 ± 4.6	161.7 ± 6.2	0.113
Gestational weeks	39.0 (39.0–40.0)	39.0 (39.0–40.1)	0.346

Data were presented as as mean ± standard deviation or median (p25, p75). Group D: dexmedetomidine group; Group C: saline control group.

**Fig. 2:**
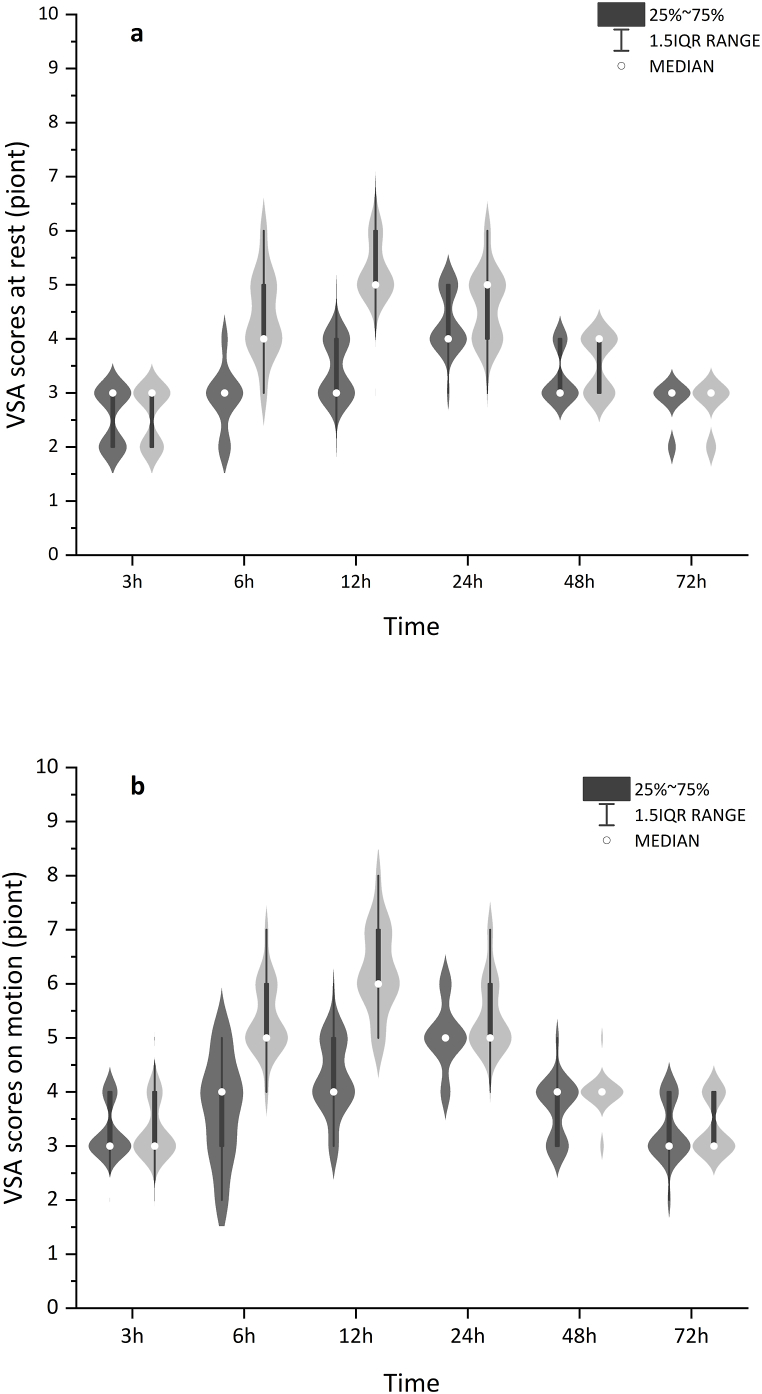
Pain scores at rest (a)or on motion (b) within 72 h were significantly lower in group D (dark grey) than in group C (light grey), *P* = 0.001 and *P* = 0.001, respectively. The box and dot plots were median (IQR) and outliers (defined as beyond 1.5 times IQR) with the violin plot indicating the density distribution of data. group D: dexmedetomidine group; Group C: saline control group.

Table 2 showed the rest and motion VAS scores in group D were significantly lower than those in group C at 6 h, 12 h, and 24 h after surgery (*P* < 0.05), with the differences at 24 h were 5.0 (5.0, 5.0)in group D versus 5.0 (5.0, 6.0) in group C (*P* = 0.04). There was a statistically significant overall trend difference between groups [*P* = 0.00, Fig. 2 (a, b)].

**Tables 2:** Visual Analog scale postoperatively at different time points.

	Group D	Group C	P-value
Rest VAS			
3 h	3.0 (2.0,3.0)	3.0 (2.0,3.0)	0.795
6 h	3.0 (2.8,3.0)	4.0 (4.0,5.0)	0.000^a^
12 h	3.0 (3.0,4.0)	5.0 (5.0,6.0)	0.000^a^
24 h	4.0 (4.0,5.0)	5.0 (4.0,5.0)	0.043^a^
48 h	3.0 (3.0,4.0)	4.0 (3.0,4.0)	0.069
72 h	3.0 (3.0,3.0)	3.0 (2.8,3.0)	0.756
Motion VAS			
3 h	3.0 (3.0,4.0)	3.0 (3.0,4.0)	0.737
6 h	4.0 (3.0,4.0)	5.0 (5.0,6.0)	0.000^a^
12 h	4.0 (4.0,5.0)	6.0 (6.0,7.0)	0.000^a^
24 h	5.0 (5.0,5.0)	5.0 (5.0,6.0)	0.042^a^
48 h	4.0 (3.0,4.0)	4.0 (4.0,4.0)	0.066
72 h	3.0 (3.0,4.0)	3.0 (3.0,4.0)	0.390

Data were presented as median (p25,p75). VAS: Visual Analog scale; Group D: dexmedetomidine group; Group C: saline control group.

a Statistically significant.

Table 3 shows the time to first PCA in group D was 12.5 (10.0, 18.0) h, which was longer than that in group C, which was 5.0 (3.9, 6.0) h, with median differences 8 h (95 % CI 6 h–11 h, *P* = 0.00). The PCA frequency within 24 h postoperatively in Group D was 3.0 (1.8, 4.3), which was less than that in Group C [5.0 (3.0, 6.5), *P* < 0.05]. The PCA frequency within 48 h postoperatively in both groups was similar (*P* > 0.05).

**Table 3:** The PCA usage.

	Group D	Group C	P-value
The PCA frequency			
24 h after surgery	3.0 (1.8,4.3)	5.0 (3.0,6.5)	0.002^a^
48 h after surgery	3.0 (1.8,5.0)	5.0 (4.0,7.3)	0.001^a^
The first time to PCA	12.5 (10.0,18.0)	5.0 (3.9,6.0)	0.000^a^

Data were presented as median (p25,p75). PCA: Patient Controlled Analgesia; Group D: dexmedetomidine group; Group C: saline control group.

a Statistically significant.

Table 4 showed the time to achieve sensory block at the T10 level was similar in both groups, with no statistically significant differences (*P* > 0.05). The sensory and motor recovery times in group D were 10.0 (8.0, 16.3) h and 5.0 (4.0, 6.0) h, respectively, which were significantly longer than those in group C, which were 2.0 (1.0, 3.3) h and 3.0 (2.0,4.0) h, respectively (*P* < 0.05).

**Table 4:** Duration of nerve blockade.

	Group D (n = 30)	Group C (n = 30)	P value
Time to T10 (min)	4.0 (3.7,5.0)	3.8 (3.0,4.5)	0.061
Time to highest level (min)	9.0 (8.0,10.0)	8.0 (7.0,9.3)	0.159
Time to sensory recovery (h)	10.0 (8.0,16.3)	2.0 (1.0,3.3)	0.000^a^
Time to motor recovery (h)	5.0 (4.0,6.0)	3.0 (2.0,4.0)	0.000^a^

Data were presented as median (p25,p75). Group D: dexmedetomidine group; Group C: saline control group.

a Statistically significant.

There were no significant differences in the time to the first ambulation, the time to the recovery of gastrointestinal function, and hospital stay between the two groups (*P* > 0.05, Table S), the side effects including the incidence of postoperative nausea and vomiting, dizziness, abdominal distension, chills, and urinary retention (*P* > 0.05, Table S).

## 4Discussion

When dexmedetomidine was introduced as an adjuvant to intrathecal anesthesia, we observed a limited duration of postoperative analgesia. To compare the effects of dexmedetomidine and an equivalent volume of saline as adjuvants to spinal-epidural anesthesia, we examined outcomes after incorporating dexmedetomidine throughout the entire procedure. Our findings revealed significantly lower VAS scores for rest and motion at 6 h, 12 h, and 24 h post-surgery, coupled with a prolonged time to the first PCA use. Importantly, there was no observable increase in adverse effects. Our study suggested that adding dexmedetomidine to spinal-epidural anesthesia could effectively relieve postoperative pain and extend the analgesic duration to 24 h following cesarean surgery.

One strategy to enhance inadequate analgesia during anesthesia involves incorporating α2 receptor agonists as adjuvants. This can be achieved through intrathecal injection of α2 adrenergic receptor agonists, which inhibit C-fiber neurotransmitters release and hyperpolarize postsynaptic dorsal neurons to provide analgesia [13,14]. This mechanism synergizes with local anesthetics that block sodium channels [15]. Moreover, α2 adrenergic receptor agonists exert a direct analgesic effect, inhibiting somatic and visceral pain, thereby prolonging analgesic duration and enhancing efficacy [16,17]. Combining dexmedetomidine with local anesthetics not only enhances spinal anesthesia but also increases intraoperative sedation and improves maternal comfort [18]. Bajwa et al.'s study demonstrated superior analgesia and sedation, resulting in better hemodynamic stability and patient satisfaction when dexmedetomidine was added to epidural anesthesia [19].

Postoperative pain in pregnant women commonly results from incisional pain during movement or visceral pulling pain. This pain typically emerges 4–6 h after surgery, peaking 12–24 h later. One study demonstrated that a combination of 5 μg of dexmedetomidine with ropivacaine for intrathecal injection reduced the median effective dose of ropivacaine from 11.4 mg to 9.4 mg [20]. Moreover, the analgesic efficacy of low-dose ropivacaine combined with dexmedetomidine was observed to relieve postoperative pain for up to 6 h after surgery [21]. Hence, we have decided to use dexmedetomidine as an adjuvant to spinal-epidural anesthesia to maximize the duration of analgesia. The use of dexmedetomidine as an adjuvant significantly prolonged the duration of analgesia [22] without increasing adverse effects [23].

Our study demonstrated that Group D, receiving an intrathecal injection of 5 μg of dexmedetomidine, had a significantly longer median time to the first PCA compared to Group C. Additionally, VAS scores in Group D were significantly lower than those in Group C at 6 h, 12 h, and 24 h, indicating that the analgesic strategy in Group D effectively provided pain relief for up to 12.5 h without the need for PCA and extended the analgesic duration to 24 h. The binding of dexmedetomidine to spinal cord dorsal horn neurons may have caused prolonged motor blockade [24,25], but this did not delay the first mobilization time. Our study was consistent with previous evidence [[26], [27], [28]], which also found no significant impact on first ambulation after a prolonged duration of motor block.

Our research demonstrated a novel and effective method for managing pain after elective cesarean surgery. This approach reduces postoperative pain, extends the duration of pain relief, and enhances overall comfort for parturients. It has the potential to improve postoperative pain management without significant negative effects. Additionally, this suggests that further enhancements to spinal anesthesia can be achieved by adjusting drug combinations and dosages, offering more possibilities for postoperative pain relief. However, the complete benefits of this pain management strategy still need evaluation. In the future, optimal dosage, catheter depth, and placement positions should be investigated to optimize postoperative spinal anesthesia effects.

Several limitations should be considered: Firstly, the study was conducted solely at a single center, raising concerns about the generalizability of the results to other settings. Secondly, adverse effects were only assessed within the initial 72 h post-surgery. To fully assess the safety of the intervention, a long-term follow-up would be necessary. Thirdly, the study exclusively compared dexmedetomidine to a control group receiving normal saline. A valuable extension would involve comparing the efficacy and safety of dexmedetomidine with other commonly utilized adjuvants for spinal anesthesia, such as ketamine or clonidine.

## 5Conclusion

In conclusion, this study indicated that adding dexmedetomidine as a supplement to spinal-epidural anesthesia during elective cesarean section can enhance the postoperative analgesia quality and extend the analgesic duration, without increasing the risk of adverse effects such as postoperative hypotension, nausea, vomiting, and shivering.

## Funding

This work was supported by the Natural Science Foundation of Universities of Anhui Province (No. KJ 2021A0278).

## Data availability statement

The raw data used in this study are available from the corresponding author upon reasonable request.

## CRediT authorship contribution statement

**Yang-yang Wu:** Writing - original draft, Formal analysis. **Zheng Fang:** Writing - review & editing. **Kun-shan Liu:** Investigation, Data curation. **Meng-di Li:** Investigation. **Xin-qi Cheng:** Writing - review & editing, Writing - original draft, Funding acquisition, Conceptualization.

## Declaration of competing interest

The authors declare that they have no known competing financial interests or personal relationships that could have appeared to influence the work reported in this paper.
