# Supplementary material for: Whole-course application of dexmedetomidine as an adjuvant to spinal-epidural anesthesia for cesarean section: A randomized, controlled trial
Source: Heliyon. 2023 Dec 10;10(1):e23534. doi: 10.1016/j.heliyon.2023.e23534 (PMC10761565; doi:10.1016/j.heliyon.2023.e23534)
Supplement: Multimedia component 1 [file mmc1.docx]

Table S : Postoperative adverse effects and outcome

|  | Group D(n=30) | Group C(n=30) | *P* value |
| --- | --- | --- | --- |
| Nausea and vomiting | 3(10.0%) | 4(13.3%) | 1.000 |
| Dizziness | 1(3.3%) | 4(13.3%) | 0.350 |
| Abdominal distension | 5(16.7%) | 5(16.7%) | 1.000 |
| Shivering | 1(3.3%) | 0(0) | 1.000 |
| Urinary retention | 0(0) | 1(3.3%) | 1.000 |
| Constipation  Gastrointestinal recovery time (h)  Time to first ambulation (h)  Hospital stay(days) | 0(0)  20.0(8.8,26.3)  13.0(10.0,15.0)  4.0(4.0,5.0) | 2(6.7%)  22.0(10.0,27.3)  15.5(8.0,17.0)  4.0(4.0,5.0) | 0.472  0.767  0.552  0.727 |

Data were presented as median (p25,p75) or n(%). Group D: dexmedetomidine group; Group C: saline control group
